# A window of opportunity for reform in post-conflict settings? The case of Human Resources for Health policies in Sierra Leone, 2002–2012

**DOI:** 10.1186/1752-1505-8-11

**Published:** 2014-07-23

**Authors:** Maria Paola Bertone, Mohamed Samai, Joseph Edem-Hotah, Sophie Witter

**Affiliations:** 1 ReBUILD Consortium & Department of Global Health and Development, London School of Hygiene and Tropical Medicine, London, UK; 2 ReBUILD Consortium, College of Medicine and Allied Health Sciences, University of Sierra Leone, Freetown, Sierra Leone; 3 ReBUILD Consortium, Reader, IIHD, Queen Margaret University, Edinburgh, UK

**Keywords:** Post-conflict, Human resources for health, Policy analysis, Window of opportunity, Sierra Leone

## Abstract

**Background:**

It is recognized that decisions taken in the early recovery period may affect the development of health systems. Additionally, some suggest that the immediate post-conflict period may allow for the opening of a political ‘window of opportunity’ for reform. For these reasons, it is useful to reflect on the policy space that exists in this period, by what it is shaped, how decisions are made, and what are their long-term implications. Examining the policy trajectory and its determinants can be helpful to explore the specific features of the post-conflict policy-making environment. With this aim, the study looks at the development of policies on human resources for health (HRH) in Sierra Leone over the decade after the conflict (2002–2012).

**Methods:**

Multiple sources were used to collect qualitative data on the period between 2002 and 2012: a stakeholder mapping workshop, a document review and a series of key informant interviews. The analysis draws from political economy and policy analysis tools, focusing on the drivers of reform, the processes, the contextual features, and the actors and agendas.

**Findings:**

Our findings identify three stages of policy-making. At first characterized by political uncertainty, incremental policies and stop-gap measures, the context substantially changed in 2009. The launch of the Free Health Care Initiative provided to be an instrumental event and catalyst for health system, and HRH, reform. However, after the launch of the initiative, the pace of HRH decision-making again slowed down.

**Conclusions:**

Our study identifies the key drivers of HRH policy trajectory in Sierra Leone: (i) the political situation, at first uncertain and later on more defined; (ii) the availability of funding and the stances of agencies providing such funds; (iii) the sense of need for radical change – which is perhaps the only element related to the post-conflict setting. It also emerges that a ‘windows of opportunity’ for reform did not open in the immediate post-conflict, but rather 8 years later when the Free Health Care Initiative was announced, thus making it difficult to link it directly to the features of the post-conflict policy-making environment.

## Introduction

In the immediate aftermath of a conflict, governments and international donors alike recognize the necessity to rapidly rebuild the health system and increase health service provision for the population, as a goal in itself as well as an entry point for peace building [1]. At this time, one of the most problematic aspects lies in striking the balance between the humanitarian aid, focused on saving lives, and the longer term development approach to health system reconstruction and strengthening, aimed at consolidating the state, providing legitimacy to the government and ensuring effective and equitable service delivery [2-4]. This balance is even more delicate as decisions taken in the early recovery period are thought to affect the long-term development of the health system, including its efficiency and equity [5]. For this reason, it is particularly useful to reflect on the policy space that exists in the post-conflict period, by what this space is shaped and how decisions are made, and about the long-term implications of those decisions. A longitudinal approach to examining policy-making going beyond the immediate recovery years is particularly needed and has been highlighted as a gap in the literature on health systems in post-conflict and fragile settings [6].

This study aims to address this gap by focusing on the development of policies and reforms around the issue of human resources for health (HRH) in Sierra Leone over the decade that followed the end of the civil war, from 2002 until 2012. It is widely recognized that HRH represent a key component of health systems, albeit an often overlooked one, especially during the rebuilding of the health system and the re-establishing of the health services after conflict [7]. Moreover, public health workers (HWs) are an essential link between the government and the population in all areas of the country, including the most remote ones, which could help develop the legitimacy of the government and demonstrate the government’s commitment to service provision and equity [8]. However, beyond the importance of health workforce reconstruction in the post-conflict period and the need to establish an effective incentive environment to recruit, retain and motivate HWs, focusing on HRH policy development may also provide a useful case study to (i) explore the pattern of reform and features of the post-conflict policy environment and (ii) verify the hypotheses suggested in relation to post-conflict policy settings. In particular, we explore whether policies developed according to ‘path-dependency’ [9] because of historical decisions made (or not made) in previous stages and linked (or not) to the post-conflict setting. Or rather, whether there was a political ‘window of opportunity’ for reform in the post-conflict period, as suggested by some [5,7,10].

In line with this aim, the focus on the study is rather on the policy choices, the ‘drivers’ and reasons of these choices, than on the evaluation of the policy outcomes^a^. We look at the trajectory taken by the HRH policy, including the official strategic documents and the practical shifts and measures introduced to address the HRH challenges over the first post-conflict decade. Our objective is to narrate the ‘policy story’ and investigate how decisions were made, which factors and actors influenced them and what defined their timing. We believe that looking at the path taken by the HRH policy trajectory can illuminate the policy-making patterns in the post-conflict period and the legacies of such decisions in the longer term.

This paper is structured as follows. The next section briefly sets the context of the health status of the population and the health system in Sierra Leone before the conflict. Then, we present the methods and some limitations of our study. The findings section begins with the health system and HRH context in the immediate aftermath of the war and then narrates the policy story, depicting how HRH policy developed from 2002 until 2012. In the discussion section, the post-conflict policy-making trajectory and its features are identified and analyzed, before concluding with a review of the research questions.

## Context

Sierra Leone emerged in 2002 from a 10-year period of war and social and economic unrest. During that time, about 50,000 people were killed and 2 million displaced, which amounted to almost half of the population. It is estimated that more than 20,000 children were conscripted as soldiers [11].

Studies carried out before the conflict provide some information on the health status of the population and on the health system. Data from the 1974 census show that life expectancy at birth was 36–40 years for females and 33–37 years for males and the infant mortality rate was 225 per 1000 [12,13]. In 1980, 31 of the 146 chiefdoms (the lower level in the administrative system in Sierra Leone) had no government health facilities, whether a hospital or a dispensary, and only 5-10% of children below the age of 5 were enrolled at a clinic [12]. According to some studies, the underutilization of health care services, particularly in rural areas, was related to the low availability of healthcare facilities, poor quality of services in the available public facilities [14], frequent drug stock-outs and irregular payment of health workers salaries [15]. As a consequence, most people chose to buy drugs from the market, visit private or mission clinics or make unofficial payments to healthcare workers in public health facilities. Against this background, user fees were introduced in the 1980s, through the Cost Recovery Policy of the Ministry of Health and Sanitation Sierra Leone emanating from the Bamako Initiative. Public health expenditure declined by 60% between 1980 and 1987, such that by 1995 91% of the health expenditure were private, of which 95% were out-of-pocket expenditures, providing no financial protection against illness [15].

The conflict lasted between 1991 and 2002 and, although it alternated between periods of higher and lower intensity and affected the areas of the country in different ways, it paralyzed the economy and the provision of public services and caused the destruction of the infrastructures and governmental institutions throughout the country. The public health system in the aftermath of the conflict was practically collapsed. Only 16% of the health centers were still functioning by 1996, mainly in Freetown [16]. Recent data paint a dire picture of the health situation in the country. Maternal mortality remains extremely high at 857 deaths per 100,000 live births for the period between 2003–2008 [17], while in 2010 under-five mortality was estimated at 217 per 1,000 live births and infant mortality at 128 [18].

## Methods

This study is part of a research project carried out by the ReBUILD Project Consortium in Sierra Leone which specifically focused on health workers incentives. The overall objectives of the project are, to document how the incentive environment has evolved after the conflict and understand what influenced the trajectory; to describe the reform objectives, mechanisms, intended and unintended consequences; and to document lessons learned (on design, implementation, sustainability and suitability to context), reflecting on how they can be used to guide future interventions. The study received ethics approval from the Liverpool School of Tropical Medicine and from the Sierra Leone Ethics and Scientific Review Committee.

The overall study design of the research project utilizes both quantitative and qualitative methods and is based on retrospective collection of data and information on the 10-year period between the end of the conflict in 2002 and the time of the research, which started in 2012. Six different tools were applied to gather data. A half-day stakeholder mapping (SM) workshop was held in October 2012 in Freetown with 23 stakeholders in the health sector in order to understand the key actors who have influenced policy and practices in HRH in Sierra Leone over the post-conflict period [19]. Subsequently, a document review was carried out, based on documents retrieved through contacts in country, as well as in journals and grey literature. A total of 76 documents were identified, of which 57 were deemed relevant for HRH issues [20]. Finally, 23 key informant interviews (KII) were conducted, in and outside Sierra Leone, between October 2012 and June 2013. Twelve of the interviewees work(ed) with the Ministry of Health and Sanitation (MoHS), 6 were NGO representatives, 4 donor representatives and 1 a technical assistant to the MoHS^b^[21]. The other three data collection methods were: routine HRH data analysis, in-depth interviews with health workers and a survey of health workers. These are not described in detail in this article as this study draws from the first three research components only^c^.

The methodology adopted reflects the difficulty of collecting original data over such a long period of time and in a post-conflict setting, where information is scarce and difficult to retrieve [22]. The combination of methods was conceived so that each could build upon the others, allowing for the collection of information to be enriched in an iterative way. For instance, the document review was helpful in order to formulate preliminary hypotheses and guide the key informant interviews, and the interviews were critical to illuminate on the gaps that had emerged in the documentary review, in particular regarding the discussions, processes and dynamics between actors, for which the documents were silent. Due to the combination of data collection methods, it was possible to compare and thoroughly triangulate findings. Similarities and discrepancies were analyzed in a reflective way to better understand why perceptions and insights differ between actors and sources. This process ensured that the methodologies are complementary and helpful in shedding light on the processes of policy-making in a comprehensive way and from different perspectives.

Despite the careful triangulation of information, our methodology and sampling present the following limitations: (i) the majority of the participants during the key informant interviews and in the group discussion for the stakeholder mapping, as well as the bulk of the documents retrieved (about half), are from the MoHS or from other governmental bodies; (ii) few documents referred to the HRH situation prior to 2009, whilst more than 50% of the documents were dated after 2011; and (iii) only few respondents were present in Sierra Leone and engaged in HRH policy-making for the period under review, and particularly during the immediate post conflict period. Those who were present for the entire time found it difficult to recall events that occurred in the immediate post conflict period and emotional and personal narratives emerge rather than organizational ones.

Although the findings section is based on the chronological narration of the HRH policy evolution and does not follow in its structure the conceptual elements of an analytical framework, the analysis is inspired by political economy and policy analysis approaches [23,24]. Drawing from these approaches, rather than looking exclusively at the policy content and implementation, our analysis focuses on the interactions between the context, including the historical legacies, the evolving formal and informal institutions and power structures; the actors, both national and external, applying ideological, political and financial pressures to decision-making; and the dynamic processes of the political system [25-28]. We use these analytical tools in a flexible manner as our analysis is not performed cross-sectionally looking at a specific moment in time, but rather covers a 10-year period. We explore, for each reform or policy stage in turn, the political processes and dynamics of change, looking at the key drivers of reform, the main actors, their roles, agendas and influences, and the formal and informal arenas in which they interacted.

## Findings: the unfolding ‘policy story’

### Immediate post-conflict context and HRH challenges

By the end of the conflict in 2002, the situation of the health system was extremely challenging. Concerning HRH, little data and documentation exist and those available are often unreliable and contradictory [29]. As one respondent noted, this reflects the fact that all actors were primarily concerned with the pressing needs of the early recovery and little time was available for the production of documents and reports, and even less for academic research.

The available information shows that the challenges faced at the time in Sierra Leone are not dissimilar to those in other post-conflict contexts [7,10,29]. The basic health infrastructure was destroyed and most services were completely disrupted, especially in the eastern and southern part of the country where most of the rebel activity took place. Health facilities were grossly understaffed as many HWs had left the country, and particularly those in the higher cadres. Other HWs were employed by NGOs or held dual positions with NGOs and the MoHS [30]. The majority of those HWs who stayed in the government service preferred to work in Freetown or in the Western Area around the capital. The data available for that period clearly indicate a significant loss of qualified HWs in the public health sector in Sierra Leone which created a gap that remained to be filled in the aftermath of the conflict. Of the 203 Medical Officers that were present in the country in 1993, only 67 remained in 2005 and of the 623 State Registered Nurses (SRN) 152 remained [31]. While the private sector employed only a small minority of the health workforce, centered in the capital, in the few years immediately after the conflict, many HWs in the public sector were working with NGOs in the governmental facilities, for which they would receive incentives and training, whether under a formal agreement with the MoHS or without. NGOs supporting public facilities also recruited and funded personnel, which was later absorbed in the MoHS payroll.

In those early years, the extreme lack of coordination between the different actors in the health system appears to be an important feature of the policy context. The term ‘chaos’ frequently emerged in the respondents’ narratives:

“What happened was, during a period of chaos, most of the NGOs were operating on their own” (KII - MoHS).

“After the war, it was complete chaos. The NGOs came and went […]. They employed the nurses directly, without even consulting the Ministry. […] They never presented any budget. But this was a war. We had to bend backwards in the Ministry” (SM – MoHS).

This highlights the fragmentation of the health system at this stage and the struggle that the government through the MoHS faced to create a system and establish control over the health workforce. However, it seems that the MoHS was able to maintain a certain leadership to start the process of reconstructing the public health system. For example, in contrast to other countries in similar post-conflict situations [6,32-34], in Sierra Leone health services were provided by public facilities and were not contracted-out to other actors of the health system. Although the choice of not adopting a contracting-out approach did not appear to be made explicitly by any of the actors but was rather the consequence of the specific context, it clearly had lasting consequences which affected the future development of the healthcare system.

### The development of formal HRH policies: 2002–2009

Against this backdrop, HRH reforms began to develop. Our findings reveal that between 2002 and 2009 the progress towards policy-making for a coherent restructuring of the health workforce was not rapid or effective. Although the challenges were correctly identified by the MoHS and potential solutions being proposed (cf. for example [30,35]), very little was happening in practice.

Relatively minor changes were introduced to improve the management of HWs in order to keep the system functioning. For instance, between 2006 and 2007, the Scheme of Service was reviewed to ensure a clearer career path and HWs started receiving allowances for housing, remote area placements, and leave [35,36]. However, the major reforms suggested in the annual presentations of the MoHS HRH Manager and in other informal MoHS documents [30,35], remained unfunded and unimplemented and the response to the HRH challenges was fragmented. At the same time, a series of broad policies and strategies were being drafted – in 2002 the *National Health Policy* (NHP) [37], followed by the *Human Resources for Health Development Plan 2004–2008*[38] and then the *Human Resources for Health Policy in Sierra Leone*[39]. Similar to other post-conflict contexts, these documents tended to remain relatively vague normative frameworks rather than operational documents to be reflected in changes at peripheral level [7,22,40]. As the most recent *HRH Policy* (2012) states, “there have been two attempts to formulate national policy to guide the development and management of Human Resource for Health in Sierra Leone […], but none was finalized or adopted for implementation” ( [41]: p.6). The lack of technical and implementation capacity within the MoHS could explain why policies remained on paper. Additionally, external agencies played a significant role in this, in particular because their mandate narrowly focused on production rather than implementation of the strategies. Some key informants pointed out to the fact that these policies were externally-driven, lacking the national ownership that would ensure their effective implementation:

“People started working on their own areas and they started developing a policy and plan and things like that […]. But it was all happening in parallel, also depending […] on the focus of donors to provide TA and funding for certain things. So I think a lot of policies applied at the beginning were definitely donor-driven. WHO said ‘you don’t have a policy on this and this. We have to develop it’ , and you’ll get it.” (KII - NGO).

The piecemeal support of the international community did not allow for the strengthening of the MoHS, especially as donors focused on ‘their’ programmes, supporting one or another department or units, undermining the overall capacity of the MoHS and creating a fragmentation within the Ministry, with long-lasting consequences [4].

Among the reasons for the delay in the adoption and implementation of major shifts in HRH policy may be the lack of clear political vision on the future of the health system more broadly. Indeed, key informants agree that in the years following the conflict, strategic policies and plans were slow to be put in place or missing altogether.

“The main issue during this time [was that] the Human Resources Strategic Plan was not adequately addressing the issues of Human Resources. Because of the absence of a strategic plan, we were just swimming with ideas […] and there was no clear direction as to what to do.” (KII – donor).

“Let me tell you something, in life when you do not have a goal you are working towards and you go purposeless, aimless, you’re slow at it.” (KII – MoHS).

The consequence of the lack of political guidance and strategic vision was a general sense of ‘purposelessness’. This resonates with the findings of the documentary review, where it emerged how fluid and uncertain policy context was, as explicitly recognized by the *HRH Development Plan 2004–2008* which states that a certain flexibility will be allowed in the proposed activities “given the current level of *uncertainty* regarding the exact nature of the reforms” ( [38]: p.80 – italics added). Obviously, the broader political dimension is important to understand the lack of strategic vision for the health sector. The government elected in 2002, which seemed to initially enjoy some support, soon lost much of its popularity given its weaknesses in terms of leadership to drive for reform, especially compared to the following administration in power from 2007 ( [4] & KII). For the HRH sector, the consequence of drafting broad policies without an overall vision on the ways to rebuild and strengthen the health system was a relatively static approach, which left little space for innovation and focused mostly on “fire-fighting”, as suggested by a respondent, i.e. tackling the most immediate issues with quick-fix solutions. The situation substantially changed with the introduction of the ‘free health care initiative’ (FHCI).

### The introduction of the FHCI: 2009–2010

In September 2009, the President of Sierra Leone, Ernest Bai Koroma, announced at a donors’ conference in London his intention to launch a reform to introduce free healthcare for pregnant women, lactating mothers and children under 5 years of age [42]. Soon after, the announcement was made in Sierra Leone to the MoHS and partners and an official launching document was drafted [43]. A few months were allowed to prepare the launch of the new policy in April 2010. Without doubt, the introduction of the Free Health Care Initiative (FHCI) is the key event that emerged from the document review and that informants consistently mentioned in their narratives about the reconstruction of the health sector.

Different factors emerge as the ‘drivers of change’ for this reform. Certainly, the health status of the population with one of the highest maternal mortality rates in the world, as well as emerging evidence of financial barriers in access to healthcare, played an important part in promoting the policy ( [44] & KII). However, even more critical seems to be the role of the President and the lead he took to include the FHCI among the government’s priorities. The political dimension of the FHCI is confirmed by the President’s direct involvement in the announcement of it as a ‘Flagship Project’, by the work done by the Strategy and Policy Unit, a very influential, high-level advisory unit in charge of promoting the presidential agenda [42], as well as in numerous interviews. Additionally, the international environment and the pressure from external actors also contributed to the decision. Indeed, free healthcare was at the time an increasingly popular reform in many African countries, supported by some of the international donors, and in particular the UK Department for International Development (DfID), which also made funding available tied to the implementation of this particular reform. As one informant stated:

“You have to have it [the FHCI] in context. I know that there was a push in 2008/2009 by Gordon Brown and he decided, DfID decided to support [the reform]. And because of DfID support, […] that is why it was able to get off. Under our government’s own resources they could not [support it].” (KII – MoHS).

The launch of the FHCI provided an opportunity for health system strengthening and to address in a more comprehensive and organic way the issues that previously were partially solved with piecemeal changes. The design and preparation of the FHCI (much more than its implementation) represented an occasion to increase and improve coordination among actors and provide a broad, common objective to all stakeholders (KII). Six Technical Working Groups were put in place, of which one focused on HRH, which held meetings weekly and were tasked with designing the necessary reforms, as well as of coordinating among the different partners [45].

With reference to HRH, the launch of the FHCI played an instrumental and catalytic role in pushing reforms. It was explicitly recognized by all stakeholders that addressing issues affecting the health workforce was critical for the success of the FHCI, for at least two reasons: firstly, HWs would have to deal with an increased workload; and secondly, in order to compensate facilities and HWs for the loss in revenues due to the end of the cost-recovery. With the inputs from the Working Group, HRH reforms started developing. The result was that, by April 2010, salaries had been increased for all HWs in technical positions. The increase was substantial, ranging from 314% for the lower grades up to 705% for the higher grades [46]. As a corollary to the salary increase, an in-depth verification and cleaning of the MoHS payroll was carried out to ensure that only legitimate staff were included and to eliminate ‘ghost workers’ [47]. Additionally, a mobile recruitment programme at district level was put in place for the fast-track recruitment of new workers and of those already volunteering in the facilities [47]. At the same time, discussions began about the introduction of a system to monitor the presence of HWs in the facilities, which was later introduced in mid-2010 when staff absence begun being monitored through the Attendance Monitoring System, and January 2011 when the Sanctions Framework was implemented [48].

Obviously, the decision-making process that led to the choice, design and implementation of these reforms was less smooth and linear that it would appear from the end results. While the creation of inter-agency working groups undoubtedly increased coordination, some issues were hidden under the surface. As one respondent recalls,

“Of course we had our Working Group meetings and we would talk, but these were the ‘big lines’. If you go to the little activities, we were not so well coordinated”. (KII – NGO).

In particular, concerns emerged around the role of the donors, their different views on FHCI and on how different components of the health system could be reorganized to provide free health services. In particular, the argument between two donors around the merits of a salary increase compared to the introduction of a performance-based financing (PBF) scheme stalled the discussion for some time. As a key informant recalls,

“These meetings [of the HRH Working Group] were completely dominated by [two donors] having their ideological fight effectively. I mean, it wasn’t just those two individuals but these meetings achieved very little, because, when these two big donors are busy having a fight, week after week after week not much else gets discussed.” (KII – TA).

In the end, while conflicting agendas and ideologies may have played a role in the decision, the choice of policy approach (i.e. the salary increase) was ultimately taken on the basis of practical feasibility. Although it was recognized that PBF would have had the advantage of improving the accountability of HWs, it was also agreed that setting up a PBF scheme would have higher transaction costs and take longer than a salary increase. This was perceived as a major disadvantage given the urgency of the launch of the FHCI (KII – donor). Moreover, after a nation-wide HWs strike which took place in March 2010 in request for higher salaries, this option became inevitable. What emerges from the analysis is that the MoHS perspective seemed to have been caught in the cross-fire of the donors’ agendas and the funding possibilities that came with donors’ support. It also appears that the corollary measures taken, such as the payroll cleaning and the introduction of the Sanctions Framework, were not only strategies to improve the HRH management and performance, but also a conditional request from the donors funding the reform, and DfID in particular, in order to “protect their investment” and “minimize risk” of misuse of their funds (KII – donor).

Several episodes confirm the influence of external actors, as well as the fragmented and ‘serendipitous’ nature of policy-making at the time. Many respondents recognized the drawbacks of the technical assistance provided, characterized by high turnover and little coordination, which resulted in the loss of institutional memory, duplications and incoherence in policy-making and implementation. This is, for instance, the case with the cleaning of the MoHS payroll which was done in 2009–2010, but had already been carried out a few years before for the entire civil service ( [49] & KII). Providing another example, some informants recalled how, despite the pressures and promises of some partners, the issue of funding the salary increase, was resolved in an “entirely coincidental” way (KII – TA), when the Global Fund’s Health System Strengthening funds became available. Interestingly, the Global Fund had not participated in the Working Group’s discussions directly and its low level of engagement contributed to creating a commonly accepted narrative around the role of donors, where DfID (contributing, over three years, about 22% of the total health salaries after the increase, but highly involved in the discussion and providing substantial, direct support to the MoHS through numerous technical assistants) took a much more central role and was able to steer critical decisions, than the Global Fund (contributing 20% of the total amount, in the initial 3 years) [50].

### HRH policy-making after the Free Health Care Initiative: 2011–2012

Beyond the urgency of the FHCI launch, the momentum for the collaboration between MoHS and partners seems diminished, if not lost, afterwards. The Working Groups are reported to meet much less regularly after the launch of the FHCI and were almost inactive by March 2013. Nevertheless, two major reforms were implemented after 2010, which in fact had been discussed or planned at the time of the FHCI design: a Performance-based Financing (PBF) scheme and a Remote Allowance for HWs working in rural posts.

While the discussion of a PBF scheme became detached from the design and the planning of the FHCI as the salary increase option was preferred, meetings for the planning of PBF continued, especially between the World Bank and the Department for Planning and Information (DPI) of the MoHS. The scheme was designed and has been implemented since April 2011. Along with the World Bank, which as the promoter and the funder of the scheme is recognized to be the driving actor for its implementation, the DPI also played a critical role and remains in charge of the operationalization of the policy. In contrast, the Department for HRH (D-HRH) which is in charge of the payroll management (which, incidentally, is supported by a different donor) is far less involved in the scheme and has surprisingly little overview of the working mechanisms of PBF. The consequence of this is a further fragmentation, not only in terms of the design of the HRH policies and the package of incentive for HWs, but also of the implementation of the PBF scheme. This has been plagued with severe delays in the payments made to the facilities, which undermine the effectiveness of the scheme and may have had negative consequences on the performance of the HWs (KII).

A similar story applies to the Remote Allowance for HWs, which was introduced in early 2012. This policy had already been discussed before the launch of the FHCI; however, it was not implemented because of the lack of resources. As further funding from the Global Fund became available, the policy was finally designed and introduced. Again, the DPI is mainly responsible for its implementation and, despite some collaboration with the D-HRH to access payroll data, there appears to be a strict division of tasks between the two departments, with little transparency in its management. As a consequence, few actors seem familiar with the mechanisms for eligibility and funding. Furthermore, the Remote Allowance currently rarely reaches the HWs that are eligible for it, due to the discontinuity of the Global Fund funding, as well as the poor communication and coordination within the MoHS (KII). The separate management of the Remote Allowance creates a further fragmentation of policies and activities, even within the MoHS.

Beyond these two major reforms (and their implementation challenges), several HRH issues remain unsolved or only partially addressed. For instance, during the preparation for the FHCI, a mobile recruitment programme had been set up. However, this remained a one-off exercise. For the routine recruitment of HWs, the establishment of a Health Service Commission (HSC) was planned to replace the Human Resources Management Office (HRMO). Despite the HSC being established by a Governmental Act in 2011 and the Commissioners being nominated, the HSC appears to be still not functional in March 2013. Similarly, pre-service training has been overlooked in the rush for the launch of the FHCI, in order to focus on aspects that it was possible to address faster (e.g., recruitment of HWs and in-service training). In-service training proliferated in an uncoordinated manner and only in early 2014 was the D-HRH of the MoHS preparing an HRH Training Plan for the next 10 years, to ensure the standardization and coordination of both pre-service and in-service training. Additionally, the role of non-financial incentives for the motivation of HWs, and in particular for those in rural postings, also emerges as largely ignored by policy-makers.

In terms of official MoHS policies, while the documents prepared before 2009 have remained mostly on paper, as described above, those approved following the launch of the FHCI, and in particular, the *Human Resources For Health Policy* and the *Human Resource for Health Strategic Plan 2012–2016*[41,51] seem to have been prepared to give an *ex-post*, official shape to the changes that had already taken place at operational level in HRH strategies.

## Discussion

### The stages of policy-making in post-conflict Sierra Leone

**Figure 1:**
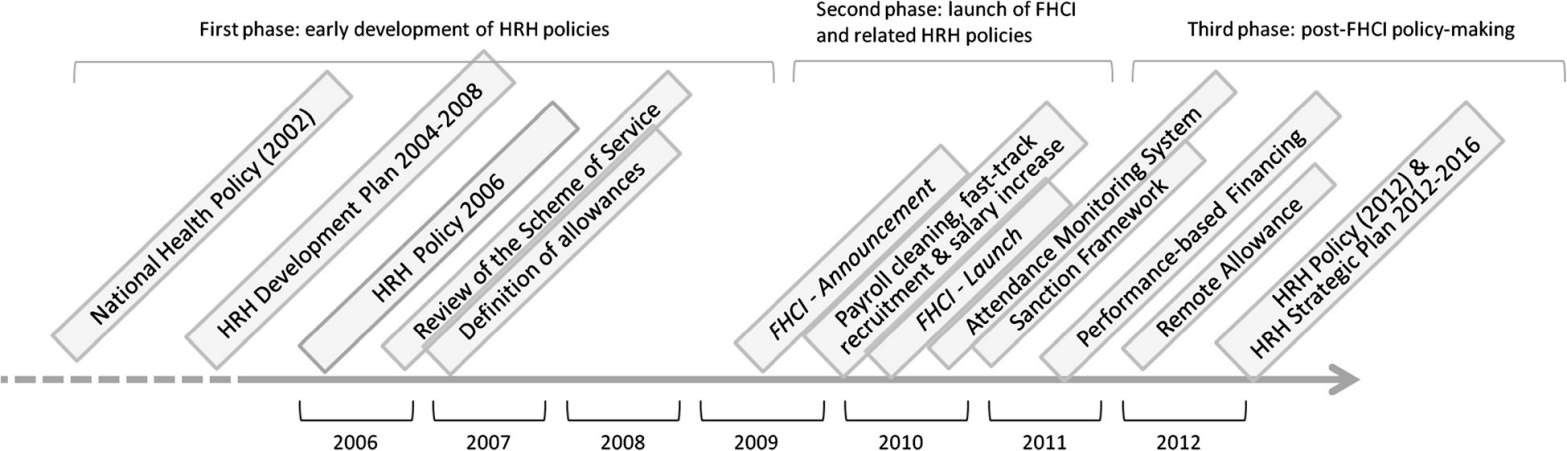
The sequencing of HRH policies and reforms in Sierra Leone: 2002-2012.

Figure 1 plots the sequence of Sierra Leone’s main HRH policy and operational reforms over time. It points out to three broad stages in the policy-making process.

The initial post-conflict period was certainly critical to define the trajectory in the reconstruction of the health system and determine the shape of the system in place. It was, for example, the decision *not* taken to contract-out health services that put the MoHS in charge not only of the stewardship of the system, but also of service delivery. The decision appears to be based on contextual factors. First, the government legitimacy was (more or less) extended to the entire country and its authority recognized by all [4]. This means that the MoHS was recognized to have sufficient capacity to reach all areas, and that public services could be provided safely without the need of delegating to third-parties. More importantly, the influence of the UK, because of the historical relations between the two countries (from the freed slaves’ settlements in Sierra Leone to the active role played by the British Army at the end of the conflict) may have led to a certain pattern in terms of aid and development. DfID preferences in terms of health systems organization may have influenced the decision to opt for direct public provision of healthcare.

However, in the immediate post-conflict, efforts to tackle HRH issues were limited to ‘fire-fighting’ measures, as noted in other post-conflict settings [4,7]. Rarely were these measures translated into formal, coherent and comprehensive Ministerial policies, as partners adopted a fragmented approach, often implemented without the involvement of the MoHS (for example, by providing salary supplementations or hiring HWs directly). Little or no opportunities opened for strategic reforms, possibly because of the uncertain political context, which is a common feature of post-conflict settings [10,22,40].

While these difficulties are generally recognized, some authors suggest that there is a ‘window of opportunity’ for reform in the immediate post-conflict period due to the political energy released by the change of regime, the fluidity of the situation with new players and ideas entering the political arena, and increased funding available [5,7,10,40]. Sierra Leone experienced a prolonged transition at the end of the conflict comparable to that of Liberia and South Sudan, rather than a ‘sudden onset’ of peace [3], but, for example in contrast to Liberia, there was no transitional government. National elections were held immediately after the peace agreement (in 2002) and the government retained a certain degree of legitimacy, control and capacity to provide services [4]. Despite these possibly favorable conditions, in those early years, there was no decision space opening for strategic health system strengthening reforms (including HRH changes), under the weak leadership of the government and the patchy interventions of the development partners. In terms of funding, the National Health Accounts reveal that the donors’ contribution to the Total Health Expenditure (THE) was 146.86 billion Leones in 2004. It then decreased to 109 billion in 2007, but substantially increased to 450.77 billion in 2010. In relative terms, this represented 18% of the THE in 2004, 12% in 2007 and 25% in 2010 [52,53]. The data confirm that, while donor funds were higher in 2004 than in 2007 both in absolute and relative terms, the substantial increase in funding followed the establishment of the FHCI.

Therefore, in the case of HRH policy in Sierra Leone, the ‘window of opportunity’ seems to have opened later than usually recognized and for reasons not necessarily linked to the post-conflict phase, but rather to the momentum created around the FHCI. Indeed, it took about eight years after the official end of the conflict for a second phase of intensive policy-making to begin, brought by strategic reforms for the health system. The disappointingly late onset and slow pace of the reconstruction process has been noted in other contexts. In South Sudan, it took three years after the peace agreement before an actual start to the recovery activities was made [3], while in Liberia the international community was not able to stimulate preparatory steps for an organic health system strengthening reform during the initial 3-year transitional phase, so that another 3 years under the new government had to go by before it was possible to start addressing the reconstruction of the health sector [54]. Also, for the case of Sierra Leone, it was a separate event, i.e. the launch of the FHCI, not related to the post-conflict setting that made it possible to overcome the political uncertainty and bring pressure for change, opening a political ‘window’ for it.

The announcement of the FHCI was the necessary instrumental event and catalyst for action in all respects of the health system, including HRH. This pattern of HRH reform is not uncommon to other contexts, whether post-conflict or not. The most salient moment in this trajectory was the introduction (for reasons mostly external to the health sector) of a broader health financing reform, not specifically focused on HRH, but which had a critical impact on the HRH reform process and was instrumental to it. While Sierra Leone has been one of the few (if not the only) country to explicitly address the link between the removal of fees and the incentives faced by HWs [46], thus making the FHCI more effective (at least, in the design), the fact that a broader health financing reform may be a helpful or even indispensable entry point for HRH reform is a key insight common to other contexts ( [22] & KII).

Undeniably, following the introduction of the FHCI, some important progress was made, at least in the design of HRH policies and likely in their implementation and impact on the health system (an evaluation of the effects of the FHCI and related reforms is currently underway). However, below the surface appearance of successful reforms, issues remained for the overall planning and, as noted in other post-conflict settings [22], different HRH-related policies were managed separately with little coordination between donors, as well as within the MoHS, between the different departments.

After the launch of the FHCI and related reforms, a new phase in HRH policy-making can be identified. In this phase, post-conflict issues and features become less apparent. Compared to the previous phase, the pace of HRH decision-making and reforms slowed down, losing the previous momentum. The Working Groups almost stopped meeting altogether and coordination became more difficult. Additionally, with reduced political pressure for the policies introduced after the FHCI, implementation of the policies has not followed the design and there are several problems and delays in their execution.

### Features of the policy-making context

The HRH policy trajectory in Sierra Leone shows the role played by historical events and contextual factors in constraining future choices (the concept of ‘path dependency’). As noted in other post-conflict countries, uncontrovertibly “the future health system [is] shaped by the present decisions” ( [22]: 665). In the case of Sierra Leone, for example, the fact that the contracting-out approach, which is often adopted in post-conflict settings, was not taken, has affected the subsequent trajectory of policy-making in HRH and beyond. However, despite the fact that some decisions appear irreversible because of how policies developed in previous stages, the Sierra Leonean HRH policy trajectory also shows that it is possible to generate radical reforms in the health sector. As pointed out in the literature, political uncertainty and (politically) fragmented health systems are unlikely to produce “big non-incremental change”. Nevertheless, the realization of propitious conditions could increase the likelihood of such change taking place [55]. In the case of Sierra Leone, the emergence of a powerful initiative, which acted as catalyst both with respect to the internal political will and the external (political and financial) support, was critical to build momentum, open a political ‘window of opportunity’ and create widespread support for radical reform in all aspects of the health system, including HRH.

It could be argued that some elements more common in a post-conflict context facilitated this process. One of these features is the fluidity of power relations and dynamics between influential actors that could facilitate reform. An example of this emerged in our study. While in other countries the professional boards are a powerful actor and the relations between those bodies and the MoH are entrenched in the system, often limiting the space for reform on HRH issues, in Sierra Leone the power relations with the professional associations seemed much more fluid. The Nursing Board, for instance, is chaired by the Chief Nursing Officer (Director of Nursing) at the MoHS, and is by definition aligned to the decisions taken by the MoHS, so that there is less or no opposition to radical changes. No opposition to the introduction of the Sanction Framework came from any of the professional boards on behalf of their affiliates (KII). Secondly, it is possible that because of the state of the health system, the launch of the FHCI could not be based on some relatively minor, incremental measure, but it required wider reforms, including for HRH. It could be hypothesized that in other non post-conflict contexts, such reforms could be postponed or diluted over time, while in a reconstruction context, the gravity of the situation, accompanied by the general climate of reform, renovation and change could foster new initiatives and gather national and international support around them. Indeed, similarly to South Africa in 1994 where the post-crisis situation created both an opportunity and a need for dramatic change [56,57], Sierra Leone has enjoyed high levels of political interest and pressure. This was coupled with substantial donor funding and technical assistance, while in other sub-Saharan Africa countries free health care initiatives were introduced without generating such momentum (as for example in Burundi, Burkina Faso, Ghana, Senegal, Sudan and others [58-64]). The reasons are likely to be related to the combination between (i) the national political conjuncture under the new government interested in implementing a visible and successful flagship reform, (ii) the international momentum around the improvement of Maternal and Child Health and the introduction of fee exemptions, as well as the major role played by some donors, and especially by a donor such as the UK with close historical ties to Sierra Leone, and (iii) the health needs of the population (in particular, with reference to the high maternal mortality levels).

Other features of the policy-making environment that our analysis highlights are less specific to the post-conflict context. It could be argued that they are not qualitatively different from those in low-income settings, but that perhaps the differences are only quantitative (i.e. same issues but worse) or, in fact, negligible. One such feature relates to the role of external actors in influencing the policy-making processes, which occurs in non post-conflict settings and is well documented in post-conflict where governments are under-resourced and weak [3,10,40,54,65]. Sierra Leone is no exception and, although evidence and health needs certainly played a role, the approach adopted for decision-making seems to be a pragmatic one, where the critical issue of the availability of funding allowed space for donor influences. Also, some HRH measures, such as the reorganization and management of the payroll, received high levels of donor-funded technical assistance, which may have allowed their realization, but raises concerns around their sustainability in the longer-term. Additionally, despite the noteworthy increase in the alignment of partners to the ministerial policies during the preparation of the FHCI, there appear to be some disconnections between the different actors. The fragmentation of views and agendas was partially overcome by the urgency to make decisions at the time of the launch of the FHCI. However, the lack of coordination became problematic later on, as the political pressure for rapid reforms was reduced. The result was fragmented policy-making, a set of policies that are not completely coherent and a largely ineffective implementation of some of those policies [10]. Moreover, reforms remained incomplete as the adoption and implementation of other necessary measures (e.g., recruitment and deployment of HWs, improved pre-service training and development of non-financial incentives) were not pursued or pursued in a slow and partial manner.

Finally, the apparent success of Sierra Leone in addressing HRH issues by taking advantage of a window of opportunity for reform cannot hide the evident challenges of having HRH changes pushed forward by a short-lived political pressure. As a consequence of the urgency of the reforms, preference was often given to one-off exercises, such as the mobile recruitment, or shorter-term solutions (as for example the decision to overlook pre-service training or the postponement of the introduction of the remote allowance). Similarly to other settings [65], much attention was generated around the design of the policies, while far less was given to their implementation at local level, which remains problematic, despite some innovative features, such as use of civil society monitors at facility level^d^.

## Conclusions

‘Post-conflict’ is a relatively little studied and poorly understood period of time, which may be extremely influential for the reconstruction of the health system after a period of social and political unrest. The trajectory of HRH policy developments in Sierra Leone provides a useful case study to examine the pattern of reform and the features of the post-conflict policy-making environment, as well as to reflect on the hypotheses about ‘path-dependency’ and ‘windows of opportunity’ in the policy-making processes.

Our analysis identifies different stages in the policy-making processes and discusses the key drivers that determined the shifts and the progression along the policy trajectory. In terms of context, it appears that policy-making was driven by the changing overall political situation, at first uncertain and later on more clearly defined as the new government set its priorities and put pressure for the success of its ‘flagship’ reform. It has also shown that the sense of need for radical change (and the decision space for it given by the evolving political dynamics) also played an important part. In terms of actors, the will of internal high-level political players, as well as the pressure of international partners contributed to the emergence of a catalyst initiative (the FHCI). Looking specifically at the decisions taken on HRH, the role of the agencies in influencing the reform options adopted emerges more clearly, given the fluidity of power relations in the health sector, as well as the relatively weak hierarchical structures and the fragmentation between departments within the MoHS. The donors’ availability of funds to support reform, but also, importantly, their direct participation in policy-making forums and the provision of technical assistance in key roles within the MoHS defined the relative capacity of these agencies to influence policy-making.

Our analysis of ‘path-dependency’ and ‘windows of opportunities’ allows reflection on the overall processes and patterns of policy change over time. ‘Path-dependency’ and the influence of the decisions taken (or not taken) in previous stages of the policy-making process contributed to define the trajectory and limit the options available. Nevertheless, the case of Sierra Leone shows that some events, by creating an alignment of actors and agendas, can act as catalyst for substantial (not incremental) change. Indeed, the pattern of HRH policy in Sierra Leone allows us to reflect on the timing of such political ‘window of opportunity’ for reform along the recovery process. As noted for other post-conflict countries, despite the potential opportunities for needed reforms to be introduced with less resistance post-conflict, “long-suffering health systems are poor reformers” ( [51]: 662). From our analysis, it emerged that the decision space for the reform of the health system did not open in the immediate post-conflict period, which was instead characterized by incremental policy-making and stop-gap measures. A window of opportunity opened later on (8 years after the end of the war), making it difficult to link it directly to the features of the immediate post-conflict policy-making environment.

## Endnotes

^a^For an assessment of the outcomes of the HRH policy making, and an analysis of the evolving incentive environment in the post-conflict period and how it affected the recruitment, retention and performance of HWs, see further work carried out by the ReBUILD Consortium (http://www.rebuildconsortium.com/publications/index.htm).

^b^Quotes from the stakeholder meeting are marked SM, while those from key informant interviews are marked KII. In both cases, the type of organization to which the respondent belongs to is also detailed (i.e., MoHS, donor, NGO, or TA), unless the same issue was mentioned by more than one respondent.

^c^Further work making use of these data is ongoing and will be available on the ReBUILD Consortium website (http://www.rebuildconsortium.com).

^d^A civil society organization, the Health for All Coalition (HAC), was entrusted in 2011 with the function of guaranteeing an independent oversight on the implementation of the FHCI and in particular to monitor the possible under-the-table payments of patients and HWs’ attendance.

## Competing interests

The author declares that they have no competing interest.

## Authors’ contributions

SW, MS and JEO designed the study. All authors participated in the stakeholder workshop. MPB and SW carried out the interviews and the documentary collection, and planned the analysis. MPB analyzed the data and drafted a first version of this article, which was commented on by all authors. All authors read and approved the final manuscript.
